# A model for ‘reverse innovation’ in health care

**DOI:** 10.1186/1744-8603-9-40

**Published:** 2013-08-30

**Authors:** Jacqueline W DePasse, Patrick T Lee

**Affiliations:** 1 Department of Internal Medicine, Massachusetts General Hospital, 55 Fruit Street, Boston, MA, USA; 2 Director, Global Primary Care Program, Massachusetts General Hospital, Boston, MA, USA

## Abstract

‘Reverse innovation,’ a principle well established in the business world, describes the flow of ideas from emerging to more developed economies. There is strong and growing interest in applying this concept to health care, yet there is currently no framework for describing the stages of reverse innovation or identifying opportunities to accelerate the development process. This paper combines the business concept of reverse innovation with diffusion of innovation theory to propose a model for reverse innovation as a way to innovate in health care. Our model includes the following steps: (1) identifying a problem common to lower- and higher-income countries; (2) innovation and spread in the low-income country (LIC); (3) crossover to the higher-income country (HIC); and (4) innovation and spread in the HIC. The crucial populations in this pathway, drawing from diffusion of innovation theory, are LIC innovators, LIC early adopters, and HIC innovators. We illustrate the model with three examples of current reverse innovations. We then propose four sets of specific actions that forward-looking policymakers, entrepreneurs, health system leaders, and researchers may take to accelerate the movement of promising solutions through the reverse innovation pipeline: (1) identify high-priority problems shared by HICs and LICs; (2) create slack for change, especially for LIC innovators, LIC early adopters, and HIC innovators; (3) create spannable social distances between LIC early adopters and HIC innovators; and (4) measure reverse innovation activity globally.

## Introduction

‘Reverse innovation’ — the flow of ideas from lower to higher income settings — is gaining traction in health care as a way to generate innovative ideas. A recent review article [1] identified examples spanning the six World Health Organization (WHO) health system building blocks [2] where developed countries benefited from ideas originating in developing countries. The *Globalization and Health* theme issue on reverse innovation, which this paper introduces, further expands this emerging literature.

**Figure 1:**
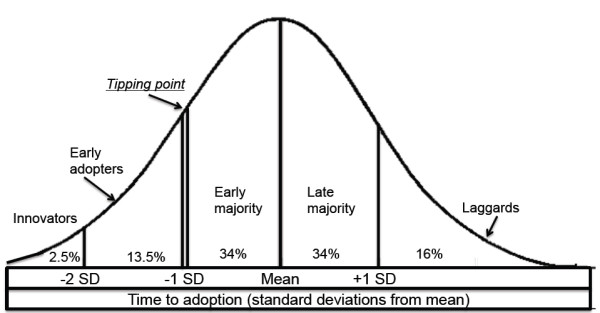
**Dynamics of innovation spread **[3]**,**[4]**.**

Our paper presents a model for reverse innovation (RI) in health care (Figure 1), drawing together core concepts from the business and innovation literature. The model describes an innovation pathway that begins with the identification of a problem common to low-income countries (LICs) and higher-income countries (HICs), an innovation’s development and spread within the LIC, its crossover to the HIC, and its spread in the HIC, thus completing the RI process.

The model is useful in three ways. First, it provides a yardstick for tracking and comparing the stage of different RI initiatives. Second, it reveals opportunities to intervene and accelerate the movement of ideas through the RI pathway. And third, it suggests metrics to track the progress of individual ideas along the RI pathway and to assess, in the aggregate, the overall maturity of the field.

We begin by summarizing core concepts from the business and innovation literature. We then describe the model for RI in health care and illustrate it using three current examples. Finally, we discuss specific actions that policymakers, entrepreneurs, health system leaders, and researchers can take to facilitate the movement of promising ideas through the RI pipeline. We hope that by encouraging this mechanism for diffusion of innovation, key stakeholders will be incentivized to invest in these technologies, which ultimately will benefit members of LIC and HIC alike.

### Core concepts from the business and innovation literature

#### ***What is reverse innovation?***

Reverse innovation refers to the process of first identifying and/or fostering a successful innovation in a LIC that addresses an unmet need in a HIC, then adapting and spreading the innovation from the LIC to the HIC. It is, in a nutshell, learning from and investing in poorer settings as one way to tackle problems in wealthier settings that require out-of-the-box solutions.

First described by Govindarajan and Trimble [3], RI can be successful because of the unique characteristics of developing countries that provide powerful incentives, or “gaps” that drive innovation. Five gaps have been described in the literature. First, the market in LICs is typically *higher volume for lower price*, or “value for many” instead of “value for money [5].” Radically different approaches are often needed to achieve satisfactory performance at ultra-low price points, providing powerful incentives to create low-cost devices of acceptable quality. Second, the physical *infrastructure* is often underdeveloped, providing a “clean slate” for rapid implementation of cutting edge products and technologies. New systems and technology can be rapidly implemented without having to overcome the resistance of convincing people to switch from existing, more familiar systems and technologies. Third, there is pressure for *sustainability* in resource-limited areas, favoring “green” solutions due to larger and more rapidly growing populations whose consumption could deplete existing natural resources and damage the local and global environment. Fourth, *fewer regulations* in many LICs allow for faster pace of innovations, which once proven in other parts of the world may be more likely to pass through regulatory bodies in HICs. Fifth, there are different *preferences* that inspire creativity in design.

In the context of health care, a sixth factor is operative in LICs: often overwhelming need, which adds urgency and a moral imperative to create effective, scalable solutions. In essence, stakeholders in LICs have more to gain from the success of a targeted innovation, providing powerful incentive to generate creative solutions.

#### ***What is needed for diffusion of innovation in health care?***

In applying the RI concept to health care, we draw upon three influential ideas from diffusion of innovation theory.

**Figure 2:**
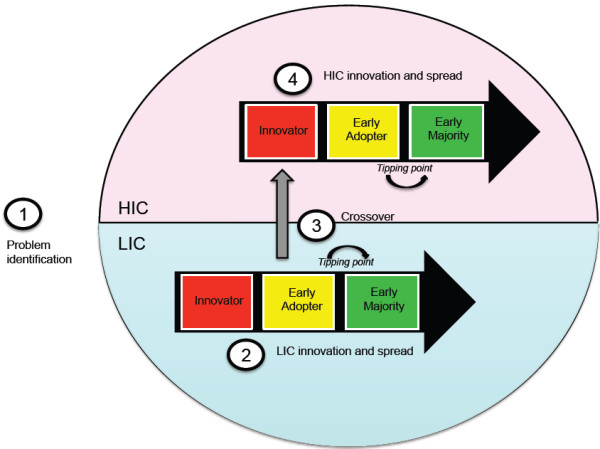
A model for reverse innovation in health care.

First, what determines the dynamics of innovation spread? Everett Rogers in his seminal work *Diffusion of Innovation*[6] divides our society into five categories based on their likelihood of adopting innovations (Figure 2):

(1) * Innovators* are the first individuals to adopt an innovation. Comprising about 2.5% of the population, innovators tend to have high social class, high risk-tolerance, financial liquidity, and close contact to other innovators. Innovation failures are most common in this group, but financial resources help absorb these losses.

(2) * Early Adopters* are the second group of individuals to adopt an innovation. Making up about 13.5% of the population, early adopters share several characteristics with innovators (high social class, financial liquidity), but they are more discrete in their adoption choices and tend to have a high degree of opinion leadership among their peers.

(3) * Early Majority* is the third adoption group. Approximately 34% of the population, these individuals tend to follow the lead of early adopters.

(4) * Late Majority* and (5) *Laggards* — the last groups to adopt innovations.

Gladwell observed that when an innovation has been adopted by 15-20% of the population, it may pass a “tipping point,” beyond which spread is likely to occur throughout the remainder of the system [7]. This threshold falls somewhere between the tail end of the early adopter group and the first part of the early majority.

Second, what influences the decision to adopt or reject an innovation? According to Rogers, ideas that are easily adoptable satisfy the following criteria; they are *better* than existing alternatives, they are *relevant* to the local context, they are *simple* and therefore easy to communicate and understand, they are *easily tested*, and they are *visible to others*.

Third, what can be done to accelerate innovation spread? Drawing from across the innovation literature, Berwick offered seven ‘rules’ to nurture innovation in his 2003 JAMA paper, *Disseminating Innovations in Health Care*[4]. Of these, he later underlined these two:

(1) * Create slack for change* — innovation takes time, money, energy, and resources as well as an openness in the industry to make changes.

(2) * Make early adopter activity visible* — spread requires social interaction, or the communication of new ideas across a “spannable social distance” from a credible source.

Core concepts from the business and innovation literature

**Why does reverse innovation work?** (Govindarajan and Trimble [3])

5 innovation gaps:

1. Price

2. Infrastructure

3. Sustainability

4. Regulations

5. Preferences

**Dynamics of innovation spread** (Rogers [6] and Gladwell [8])

1. Innovators (2.5% of population) solve the problem.

2. Early adopters (13.5% of the population) identify and endorse the solution, driving its spread.

3. A tipping point occurs with adoption by 15-20% of the population, around the transition point from the early adopters to the early majority.

**What drives adoption of an innovation?** (Rogers [6])

5 factors:

1. Better

2. Relevant to local context

3. Simple

4. Easily tested

5. Visible to others

**How do we accelerate innovation spread?** (Berwick [4])

1. Create slack for change

2. Make early adopter activity visible

### A model for reverse innovation in health care

We integrated the above concepts to create a new model for reverse innovation in health care (Figure 1). This model specifically explores the ‘crossover’ point where ideas begin to transition between two distinct innovation curves: the first originating in a LIC, and the second occurring later in a HIC. The model has four steps: (1) problem identification; (2) LIC innovation and spread; (3) crossover; and (4) HIC innovation and spread.

#### ***Step 1: problem identification***

The pathway for RI in health care begins with the identification of a high-priority problem that is: (1) common to both LICs and HICs, and (2) subject to more favorable innovation conditions in the lower-income setting. Examples of such problems range from the need for low-cost and user-friendly diagnostics, to mobile health information technology solutions, to the need to advance the effectiveness of inter-professional teams to enable close-to-client delivery of health services. The five “innovation gaps” of price, infrastructure, sustainability, regulations, and preferences drive innovation by creating a favorable environment for new ideas to flourish. Whereas “innovation” alone begins with local problem identification, RI begins with identifying common problems, thus opening the possibility for new forms of collaboration between stakeholders in LICs and HICs.

#### ***Step 2: low-income country innovation and spread***

Once a problem is identified, a sound solution must be piloted by innovators and taken up by early adopters in a LIC. Given the urgency of need and large gap between needed and available care, sound healthcare innovations will quickly spread in these settings. Creating slack for change (in the form of time, money, personnel, etc.) for LIC innovators to develop and test new ideas, and then share these solutions with LIC early adopters can accelerate this vital step. The vetting process by early adopters can help improve the soundness of the solutions, as measured against Rogers’ five factors: that the innovation is better, relevant, simple, easily tested, and visible to others.

#### ***Step 3: crossover***

In order for RI to occur, the idea must cross-pollinate from lower- to higher-income settings. Whereas innovation spread *within settings* moves from early adopter to early majority populations (as described above by Rogers in diffusion of innovation theory), we argue that spread *across settings* is more likely to move from LIC early adopters to HIC innovators. This is a variation of Berwick’s *make early adopter activity visible* rule: here, it is the HIC innovators who need to form spannable social distances with LIC early adopters. To accelerate the crossover step, new venues for social interaction — in-person, virtual, synchronous, etc. — could be developed, with additional slack provided to both LIC early adopters and HIC innovators to foster transfer and uptake of new ideas.

#### ***Step 4: high-income country innovation and spread***

Once an idea has crossed over, it must then take hold in the HIC. Rogers’ factors and Berwick’s rules apply, but the specifics of what constitutes better, relevant, simple, easily tested, visible to others, or the slack necessary for change will all require recalibration for the new context. For example, proof of superiority to existing technology or standard of care in a clinical trial may be necessary for uptake by HIC early adopters, and more rigorous comparative and cost-effectiveness data may be needed to convince the HIC early majority to change practice. Resources must be allocated not only to support the innovation and its spread, but also to repurpose existing personnel or capacity that may be made obsolete by the new method. LIC innovation spread may also be relevant: if the idea has ‘tipped’ into the LIC early majority, then HIC early adopters may be more likely to take it up.

### Three examples of reverse innovation

We use three current RI examples to illustrate our model (Table 1), one each from the WHO health system building blocks of medical products, health information, and service delivery. These three are real-world examples that demonstrate the Reverse Innovation pathway in action:

**Table 1:** Three examples and four steps of reverse innovation in health care

**Example and WHO health system area**	**Step 1. Problem Identification**	**Step 2. LIC innovation and spread**			**Step 3. Crossover**	**Step 4. HIC innovation and spread**
		**Innovation**	**Description**	**LIC spread**		
**1) Medical products, vaccines, and technologies**	Need for low-cost, rugged, portable health diagnostics for use in resource-limited areas by non-specialist personnel	General Electric’s MACi EKG machine [9], developed in partnership with Indian leaders at GE for rural health clinics in India	Price point = $550 USD, >10 times less than standard EKG machines. Additional features: lightweight, durable, minimalist easy-to-use interface.	Viewed as a commercial success by GE leadership (No publically-available data on number of units sold)	Success in India prompted GE to develop MAC 600 and MAC 800, adaptations of the simple EKG machine for value-oriented US consumers	The slightly more sophisticated version was sold to primary care clinics around the US (no publically-available data on number of units sold)
**2) Health information**	Need for gathering and sharing real-time information to map the impact and response to natural and man-made disasters	Ushahidi [10], developed in the aftermath of the 2008 Kenyan presidential election as a way to map eyewitness reports of violence	Uses crowdsourcing to gather critical and timely information from smartphones and map them in a central database	>50 projects in LIC countries ranging from mapping Zimbabweans’ opinions on door-to-door HIV testing to finding victims of Haiti’s 2010 earthquake	Recognition that crowdsourcing approach could be readily applied in HICs	US and Europe examples include: used in New Orleans to report health hazards and chemical spillages during hurricanes; used to promote situational awareness during the 2012 London Olympics
**3) Service delivery**	Need to provide close-to-client services and address underlying social determinants of health in resource-limited areas	Partners In Health (PIH) [11] community health worker (CHW) and wraparound service delivery model, first applied to HIV patients in rural Haiti	CHWs visit patients at home, help overcome barriers to care, and provide psychosocial support. Food, transport, and housing support directly address root causes of disease.	Used by PIH in range of LICs and adopted by many others. Likely has passed tipping point, i.e., 2012 multinational campaign to train and recruit one million CHWs in Africa [12].	Adapted to poor urban US populations by innovative PIH team, as the Prevention and Access to Care and Treatment (PACT) program [13].	Among HIV-positive patients in Boston, PACT reduced inpatient hospital stays by 35% and decreased hospital costs by nearly 50% [13]. PACT’s success influenced similar models by other US innovators, including Iora Health [14] and Transitions Clinic [15].

**Figure 3:**
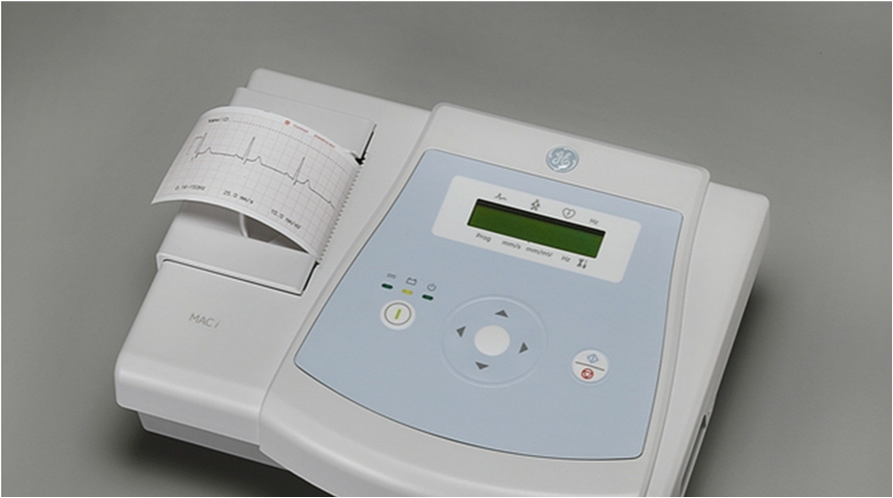
**General electric MACi ECG machine **[7]**.**

1. *Medical products* — General Electric’s low-cost MACi EKG machine [7] was developed after identifying a need for a durable, portable, low cost alternative to the current EKG technology in rural India. Researchers in Bangalore created this tool specifically for the LIC conditions, and after early adopter success the EKG was modified and adapted for US value-oriented customers [9] (Figure 3).

2. *Health information* — Ushahidi, a crowdsourcing program used to map disaster impact and response was first used during the 2008 Kenyan presidential election and subsequently applied in the aftermath of the 2010 Haiti earthquake. The widespread use of mobile phones and lack of pre-existing infrastructure in Kenya and Haiti created “gaps” or ideal conditions for this technology. After success in these LIC settings, Ushahidi “crossed over” for use in the US state of Louisiana to monitor infrastructure damage during hurricanes [10].

3. *Service delivery* — Partners In Health is a close-to-client delivery model which was pioneered in rural Haiti that uses community health workers and wraparound services to address underlying social determinants of health [11]. Geographic barriers to conventional models and lack of existing medical infrastructure in Haiti provided the demand for innovation. The concept was successful in this low resource setting and was subsequently adapted to the US in the slightly modified PACT program.

Though existing formal research is limited, readers interested in a review of current RI examples across the six WHO health systems building blocks may wish to read further in this *Globalization and Health* series and reference the review by Syed et al. [1]. Additional examples may be found in Govindarajan and Trimble’s work “Reverse Innovation.”

### Specific actions to accelerate the reverse innovation pipeline

“Skate to where the puck is going to be, not where it has been.” – Wayne Gretzky

There is no shortage of need or opportunity to leverage RI in health care. It is the proverbial ‘open ice’. Forward-looking policymakers, entrepreneurs, health system leaders, and researchers may wish to gain first-mover advantage in this nascent field. Interestingly, the RI paradigm aligns the incentives of wealthy actors who need out-of-the-box solutions to intractable problems and poorer settings where innovation conditions are more favorable. Yet where to intervene? How do we accelerate the movement of innovations through the RI pipeline?

We offer four recommendations, with specific actions for policymakers, entrepreneurs, health system leaders, and researchers:

Recommendation 1 — identify high-priority problems shared by lower and higher-income countries

• Policymakers and health system leaders could jointly commission a WHO-led “global to local” initiative to identify high-priority problems that could be addressed using RI solutions. Alternatively, this could be done at the national level through organizations such as the Institute of Medicine (IOM) in the United States or the National Institute for Health and Clinical Excellence (NICE) in the United Kingdom.

• Entrepreneurs could develop a website with three main functions: (1) facilitating RI problem identification by patients, providers, and health system leaders; (2) providing a searchable database of promising RI solutions; and (3) creating a marketplace for potential funders and innovators to convene.

Recommendation 2 — create slack for change, especially for LIC innovators, LIC early adopters, and HIC innovators

• Entrepreneurs, foundations, and universities could jointly organize a global Grand Challenges in Reverse Innovation competition, to boost visibility and award seed funding to enable the best RI solutions to move through the ‘LIC innovation and spread’ and ‘crossover’ steps of the RI pathway*.*

• Researchers could characterize and incentivize the most promising ideas at the LIC innovator and early adopter stages through foundation and NIH funding, creating the necessary benchmarks to compare such innovations across quality, cost, and impact.

• Entrepreneurs could invest directly in the best RI ideas through an online marketplace of ideas, accelerating their maturation from the innovator to early adopter stage in LICs, as well as their crossover to HICs.

• Health system leaders could create RI zones where outside ideas could be efficiently pilot-tested (fulfilling Rogers’ ‘easily tested’ and ‘visible to others’ factors), thereby lowering the barrier to adoption and facilitating crossover from LICs to HICs.

Recommendation 3 — create spannable social distances between LIC early adopters and HIC innovators

• Policymakers, entrepreneurs, and health system leaders could create new venues for social interaction between LIC early adopters and HIC innovators. These could include synchronous channels, such as conferences, learning collaboratives, or new roles for innovators to rotate through lower income settings to observe, compare, and select appropriate ideas for pilot testing. They could alternatively include asynchronous channels, such as email distributions, brief online videos (e.g., TED-Reverse Innovation), or website blogs.

• Researchers and entrepreneurs could team up as HIC innovators, leveraging existing infrastructure for global academic collaboration to discern the most promising LIC solutions, pilot test them in HICs, and generate the necessary evidence for uptake by HIC early adopter populations.

Recommendation 4 — measure reverse innovation activity globally

• Researchers could use the four-step model for RI in health care to characterize the state of global RI activity. We hypothesize that a plurality of high-potential RI solutions are currently trapped in *Step 2: LIC innovation and spread* due to insufficient resources to advance from the LIC innovator to LIC early adopter stage, or the absence of spannable social distances to enable crossover to HIC innovators.

## Conclusions

Many intractable health care problems in wealthy countries may be more readily solved in developing countries. This alignment of HIC need and LIC opportunity will continue to drive proliferation of reverse innovation solutions. Drawing from core concepts in the business and innovation literature, we present a model for reverse innovation in health care with useful implications for accelerating the movement of promising solutions through the RI pipeline.

We offer four recommendations for forward-looking policymakers, entrepreneurs, health system leaders, and researchers who wish to promote reverse innovation in health care: (1) identify high-priority problems shared by HICs and LICs; (2) create slack for change, especially for LIC innovators, LIC early adopters, and HIC innovators; (3) create spannable social distances between LIC early adopters and HIC innovators; and (4) measure reverse innovation activity globally.

## Abbreviations

LIC: Low-income country; HIC: Higher-income country; WHO: World health organization; RI: Reverse innovation; IOM: Institute of medicine; NICE: National institute for health and clinical excellence.

## Competing interests

The authors declare that they have no competing interests.

## Authors’ contributions

Both authors equally and substantively contributed to the ideas, research, and writing of this manuscript. All authors read and approved the final manuscript.
